# The Aging Slopes of Brain Structures Vary by Ethnicity and Sex: Evidence From a Large Magnetic Resonance Imaging Dataset From a Single Scanner of Cognitively Healthy Elderly People in Korea

**DOI:** 10.3389/fnagi.2020.00233

**Published:** 2020-08-12

**Authors:** Yu Yong Choi, Jang Jae Lee, Kyu Yeong Choi, Eun Hyun Seo, IL Han Choo, Hoowon Kim, Min-Kyung Song, Seong-Min Choi, Soo Hyun Cho, Byeong C. Kim, Kun Ho Lee, for the Alzheimer’s Disease Neuroimaging Initiative

**Affiliations:** ^1^Gwangju Alzheimer’s Disease and Related Dementias (GARD) Cohort Research Center, Chosun University, Gwangju, South Korea; ^2^Biomedical Technology Center, Chosun University Hospital, Gwangju, South Korea; ^3^Department of Neuropsychiatry, Chosun University School of Medicine and Hospital, Gwangju, South Korea; ^4^Department of Neurology, Chosun University School of Medicine and Hospital, Gwangju, South Korea; ^5^Department of Neurology, Chonnam National University Medical School and Hospital, Gwangju, South Korea; ^6^Department of Biomedical Science, Chosun University, Gwangju, South Korea; ^7^Dementia Research Group, Korea Brain Research Institute, Daegu, South Korea

**Keywords:** normal aging, aging slope, norm, ethnic difference, sex difference

## Abstract

The aging of the brain is a well-investigated topic, but existing analyses have mainly focused on Caucasian samples. To investigate brain aging in East Asians, we measured cortical and subcortical volumes from magnetic resonance imaging (MRI) scans of 1,008 cognitively normal elderly Koreans from the Gwangju Alzheimer’s and Related Dementia cohort and 342 Caucasians from the Alzheimer’s Disease Neuroimaging Initiative (ADNI) database. To determine whether the aging effect varies with ethnicity and sex, beta coefficients of age and confidence intervals (CIs) were estimated in each ethnicity–sex group using a bootstrap method and a regression analysis using the relative volume to intracranial volume as predicted. The betas or aging slopes largely were not significantly different between ethnicity and sex groups in most types of brain structures. However, ethnic differences between the two female groups were found in the brain, most cortical regions, and a few subcortical regions. Ethnic differences in brain aging are likely due in large part to genetic factors; thus, we compared carriers and non-carriers of a gene relevant to longevity and neurodegenerative diseases, such as *apolipoprotein E* (*APOE*) ε4. The regions with ethnic differences in women also showed significant differences between Korean *APOE* ε4 non-carriers and Caucasian *APOE* ε4 carriers. Furthermore, Caucasian women showed significant *APOE* ε4 effects in the largest number of regions. These results illustrate that much of the ethnic differences in females may be explained by synergistic effects of ethnic background and *APOE* ε4 carrier status. Our results suggest that sex-dependent differences of aging between ethnic backgrounds may be due to ethnicity-dependent effects of genetic risk factors, such as *APOE* ε4. We also presented the normative information on volume estimates of the brain structures of the elderly Korean people in the subdivided age groups. This normative information of the aging brain stratified by ethnicity provides the age-related reference ranges quantified to replace visual judgment and facilitate precise clinical decision-making.

## Introduction

The world’s population is aging rapidly as people live longer than before. According to the international population reports (He et al., 2016), the elderly population aged 65 and over is 617 million people and 8.5% of the total population in 2018. By 2050, the elderly population is projected to nearly triple to 1.6 billion worldwide. The aging brain becomes a very important and focused research topic. The human brain has been assessed non-invasively and so massively since magnetic resonance imaging (MRI) became popular. Early imaging studies identified the general trend that the brain shrank with advancing age, and the ventricle expanded, and additionally found regional variations in the effects of aging on the brain (Resnick et al., 2003; Scahill et al., 2003; Sowell et al., 2003).

The aging brain is a well-investigated topic. However, most researchers did not consider ethnic backgrounds and studied a sample of Westerners with a high percentage of Caucasians. Existing findings are mainly a reflection of Caucasians (Resnick et al., 2003; Scahill et al., 2003; Sowell et al., 2003; Ledig et al., 2018). Furthermore, in recent years, more and more studies are increasingly dependent on only a few data sources that are virtually monopolistic, such as the Alzheimer’s Disease Neuroimaging Initiative (ADNI; Weiner et al., 2015; Ledig et al., 2018). Asia now accounts for more than half of the world’s elderly population, and the proportion is expected to increase to two-thirds by 2050 (He et al., 2016). Particularly, the aging brain of East Asians has not been fully studied. It is not clear whether there are ethnic differences in the aging brain.

The study aimed to present the normative information of the aging change of the brain, including cortical and subcortical volumes, and to determine which factors, such as ethnicity and sex, affect the volumes in normal elderly people. For this purpose, we recruited 1,008 cognitively normal elderly people with brain MRI at the Gwangju Alzheimer’s and Related Dementias (GARD) cohort in the South Korea. The subjects were examined on only a single MR scanner to reduce inter-scanner variability of the brain images. Our MRI dataset could provide one of the most definite illustrations of the aging process of the human brain. For a Caucasian sample, we collected brain images from the ADNI dataset, which was, however, from multiple centers. Because the ADNI datasets were from diverse scanners and the resulting imaging protocols were different from ours, the absolute volume measures could be biased from each other. Thus, subjects should be used as their own control as much as possible. We analyzed the two datasets separately and quantified volumes and aging slopes at each race or sex level. Cortical and subcortical volumes relative to their intracranial volumes were used for analysis rather than absolute volumes.

We evaluated ethnic differences in cortical and subcortical volumes and their aging slopes. Our hypothesis was that ethnic differences in brain aging were due in large part to a gene relevant to longevity and neurodegenerative diseases, such as *apolipoprotein E (APOE*; Bonomini et al., 2010; Reinvang et al., 2013). The rationale was as follows: (1) Ethnic differences were from different genetic backgrounds. (2) *APOE* ε4, a major genetic risk factor for degenerative brain diseases (Shi et al., 2017; Sepulcre et al., 2018), is more popular in Caucasians (*APOE* ε4 carriers = 29.2 and 24.2% for women and men, respectively) than in Koreans (17.0 and 17.6%, respectively). (3) The odds ratio (OR) and the carrier ratio of *APOE* ε4 vary with age, sex, or ethnicity (Farrer et al., 1997).

We discovered ethnic differences of aging slopes in women, compared the *APOE* ε4 carriers and the non-carriers in them, and showed that the ethnic differences were partly due to *APOE* ε4 effects enhanced in women of a certain ethnicity. We also investigated the normative information on volume estimates of the brain of the elderly people in the subdivided age groups. This normative information of the aging brain stratified by ethnicity provides the age-related reference ranges needed to facilitate research and precise clinical decision-making close to personalized diagnosis of neurodegenerative diseases.

## Materials and Methods

### Participants

The study protocol was approved by the institutional review board of Chosun University Hospital, South Korea. All volunteers or the next of kin of patients gave written informed consent before participation. They were registered at the GARD cohort in the South Korea, from April 2014 to March 2017. One-thousand and eight cognitively normal elderly people (CN) aged 65–85 years were included in this study. All participants were evaluated by comprehensive interviews, neurological examinations, and neuropsychological tests. Neuropsychological tests consist of the Korean version of Mini-Mental State Examination (K-MMSE; Folstein et al., 1975), Clinical Dementia Rating (CDR; Morris, 1993), and Seoul Neuropsychological Screening Battery (SNSB; Kang et al., 2012). SNSB is a comprehensive neuropsychological test that assesses five cognitive domains: attention, language, visuospatial function, memory, and frontal/executive function (Kang et al., 2012). The exclusion criteria for all subjects were presence of a focal lesion on brain MRI, history of head trauma, or psychiatric disorders that could affect their mental function. Individuals with minor medical abnormalities were included.

The CN participants were selected based on two exclusion criteria: (i) their K-MMSE score is <19 points; and (ii) people with cognitive impairment in one or more domains among the five cognitive domains of SNSB were excluded. The criterion for cognitive impairment was a standard *z* score <−1.5. For diagnosis, we used the standard *z* score for each cognitive domain that was standardized in a normative sample of 1,067 elderly people ranging in age from 45 to 90 years (Kang et al., 2012).

To investigate ethnic difference, we collected Caucasians excluding Hispanics (342 CN cases) from the ADNI database^1^. For image quality, their brain MRI images were selected with a slice thickness <1.2 mm. The age range was matched with our dataset (65–85 years). Table 1 describes the socio-demographic information of the Korean and Caucasian subjects.

**Table 1:** Cohort sizes and demographics for the Korean and Caucasian datasets.

Cohort	Subjects		Age		MMSE		Education		*APOE*	
	Sex	*n*	Mean (y)	SD	Mean	SD	Mean (y)	SD	*n*	ε4 (%)
Korean (GARD)	F	638	72.1	4.3	27.1	2.2	8.4	4.1	534	17.0
	M	370	74.3	5.0	27.8	1.6	12.2	4.0	296	17.6
Caucasian (ADNI)	F	163	74.5	4.8	29.2	1.1	15.5	2.6	161	29.2
	M	179	75.6	5.2	29.0	1.1	17.0	2.6	179	24.6
*Two-way ANOVA (ethnicity, sex)*			*F*	*p*	*F*	*p*	*F*	*p*	*F*	*p*
Ethnicity			54.9	<0.001	235.1	<0.001	778.1	<0.001	13.8	<0.001
Sex			48.6	<0.001	27.3	<0.001	235.8	<0.001	0.2	0.659
Ethnicity*sex			3.5	0.063	15.5	<0.001	24.7	<0.001	0.9	0.323

*GARD, Gwangju Alzheimer’s & Related Dementia, Korea; ADNI, Alzheimer’s Disease Neuroimaging Initiative; MMSE, mini mental state examination; *APOE*, apolipoprotein E gene; F, female; M, male; y, years*.

### MRI Acquisition

We recruited a large number of Koreans (*n* = 1,008) and acquired sub-millimeter resolution brain images at a single MR scanner to minimize variances between brain images. Since there are no sub-millimeter resolution images in the ADNI dataset, we selected near-millimeter resolution images of Caucasian brains, which were scanned at multiple centers.

For Koreans, three-dimensional (3D) sagittal brain images (MPRAGE; *TR* = 2,300 ms; *TE* = 2.143 ms; *TI* = 900 ms; *FA* = 9°; *FoV* = 256 × 256; matrix = 320 × 320; slice thickness = 0.8 mm) were acquired at the 3.0 T MR scanner (Skyra, Siemens; 20-channel head coil) at Chosun University Hospital, Gwangju, South Korea. For Caucasians, 3D brain images with 1.2 mm or less slice thickness were acquired by diverse manufacturers and scanner models. The MRI scanner protocols were described in detail according to each scanner model at the ADNI site^2^.

### Measurement of Cortical and Subcortical Volumes

The volumes of cortical and subcortical structures were measured from each brain image using the standard recon-all processing pipeline of FreeSurfer version 5.3.0, which is documented and available for download online^3^. Briefly, the steps of the process include automated Talairach transformation, segmentation of the subcortical white matter and deep gray matter volumetric structures (including hippocampus, amygdala, caudate, putamen, and ventricles; Fischl et al., 2002), intensity normalization (Sled et al., 1998), segmentation of the gray matter (GM), white matter (WM), and cerebrospinal fluid (CSF), and surface modeling for the GM/WM and GM/CSF borders (Dale et al., 1999; Fischl et al., 2001). Once the cortical models are complete, a number of deformable procedures can be performed for further data processing and analysis including surface inflation (Fischl et al., 1999a), registration to a spherical atlas that utilized individual cortical folding patterns to match cortical geometry across subjects (Fischl et al., 1999b), parcellation of the cerebral cortex into units based on gyral and sulcal structure (Fischl et al., 2004; Desikan et al., 2006), and creation of a variety of surface-based data including maps of curvature and sulcal depth. This method uses both intensity and continuity information from the entire 3D MR volume in segmentation and deformation procedures to produce representations of cortical thickness, calculated as the closest distance from the GM/WM boundary to the GM/CSF boundary at each vertex on the tessellated surface (Fischl and Dale, 2000). To acquire consistent brain measures, we used the Desikan–Killiany–Tourville (DKT) atlas (Klein and Tourville, 2012) that has advantages where the regional definitions are unambiguous, and the boundaries are well suited to the FreeSurfer classifier algorithm.

### *APOE* Genotyping

Genomic DNA samples of Koreans were extracted from peripheral blood leukocytes that were isolated from whole blood collected in ethylenediaminetetraacetic acid (EDTA) tubes. The samples were genotyped using a genome-wide genotyping array (Affymetrix Axiom^®^ KORV1.0, Santa Clara, CA, USA), which was designed and optimized for Korean content by the Center for Genome Science, Korea National Institute of Health, South Korea (Jee et al., 2016). The genotyping was performed at DNALink (Seoul, South Korea). *APOE* genotypes were derived from allelic combinations of rs7412 and rs429358, which are included in the genotyping array.

### Statistical Analysis

Aging slopes, or annual percent changes, for cortical and subcortical volumes were calculated using the general linear model in R software version 3.5.3^4^. The model was adopted to be linear because the age range was narrow for our subjects, and the relationship between age and gray matter volume is nearly linear from adulthood (Sowell et al., 2003; Fox and Schott, 2004; Fjell et al., 2009b; Salthouse, 2011). The linear regression model analyses were performed controlling for sex, education, and intracranial volume on a large single-center sample (*n* = 1,008) from the GARD cohort. To find standard errors (SEs) of aging slopes, bootstrapping was used (Davison and Hinkley, 1997). A bootstrap was performed with 10,000 iterations.

In Table 2, the linear regression model analyses were performed using age, sex, ethnicity, education, and intracranial volume as predictors on each volume from both the GARD and ADNI databases (*n* = 1,350), and the Bonferroni correction as the most conservative approach to the multiple comparison problem was applied across 15 brain regions, resulting in a *p* threshold of 0.003 (*p* uncorrected = 0.05/15; *p* corrected = 0.05).

**Table 2:** Regression results using each volume of cortical and subcortical structures as the predicted variable (*N* = 1350).

	Age		Sex		Ethnicity		Education		ICV		Model
	*b*	*p*	*b*	*p*	*b*	*p*	*b*	*p*	*b*	*p*	*R*^2^
Brain volume	**−0.30**	1.1E-84	−0.03	1.1E-01	**−0.32**	1.0E-66	0.00	9.6E-01	**0.87**	1.9E-297	**0.752**
Cortical volumes	
Frontal	**−0.18**	1.4E-22	0.00	8.7E-01	**−0.43**	8.3E-73	0.02	5.2E-01	**0.73**	1.5E-163	**0.589**
Temporal	**−0.31**	2.5E-57	0.02	3.9E-01	**−0.46**	1.3E-83	−0.02	3.7E-01	**0.65**	6.2E-141	**0.597**
Parietal	**−0.18**	8.1E-24	*−0.05*	2.1E-02	**−0.62**	9.1E-142	0.02	3.5E-01	**0.63**	1.2E-137	**0.609**
Occipital	**−0.25**	2.4E-32	0.03	2.5E-01	**−0.47**	2.9E-68	0.02	4.9E-01	**0.53**	5.2E-80	**0.468**
Cingulate	**−0.14**	8.2E-11	0.01	6.9E-01	**−0.38**	1.8E-45	0.04	1.6E-01	**0.64**	5.9E-108	**0.459**
Insular	*−0.06*	8.8E-03	*0.07*	1.8E-02	**−0.33**	5.1E-35	0.00	9.5E-01	**0.62**	4.2E-98	**0.437**
Subcortical volumes	
Ventricle	**0.34**	2.7E-50	−0.04	2.1E-01	**0.08**	1.6E-03	**−0.02**	5.2E-01	**0.51**	2.8E-68	**0.420**
Thalamus	**−0.18**	3.7E-15	−0.05	1.1E-01	**−0.49**	8.9E-65	0.01	6.7E-01	**0.49**	2.4E-62	**0.386**
Putamen	**−0.25**	2.8E-22	−0.03	3.0E-01	−0.01	7.4E-01	0.04	2.3E-01	**0.41**	3.4E-35	**0.203**
Hippocampus	**−0.42**	1.1E-67	−0.01	7.8E-01	**−0.38**	2.7E-41	0.03	3.0E-01	**0.27**	3.4E-21	**0.371**
Caudate	*−0.06*	1.6E-02	**−0.12**	1.9E-04	**0.09**	1.8E-03	0.02	4.9E-01	**0.52**	1.2E-56	**0.243**
Amygdala	**−0.32**	7.4E-38	**0.12**	1.7E-04	**−0.37**	4.2E-35	0.03	3.6E-01	**0.27**	8.0E-19	**0.305**
Pallidus	0.04	1.0E-01	−0.01	6.8E-01	**−0.12**	8.8E-05	−0.03	4.4E-01	**0.43**	2.5E-36	**0.164**
Accumbens	**−0.33**	3.5E-33	0.06	1.0E-01	*0.09*	5.0E-03	*0.07*	4.5E-02	**0.14**	3.4E-05	**0.144**

*Age, sex, ethnicity, education, and intracranial volume (ICV) were used as predictors in the regression analyses. *p* indicates statistical significance. *b* indicates the standardized regression coefficient that is presented in italic at the 95% significance level, or in bold after Bonferroni correction*.

### Availability of Data

The GARD data that support the findings of this study are not openly available yet. Until we are ready to share the data publicly, the data could be available from the corresponding author (BK or KL), upon reasonable request.

## Results

### Cortical Volumes and Socio-demographic Effects

As shown in Table 2, the brain volume was significantly affected by age (*b* = −0.30; *p* < 0.003) and ethnicity (*b* = −0.32; *p* < 0.003), but not by sex and education. The brain shrank with increasing age, and the Caucasian brain was smaller than the Korean brain.

Cortical gray matter volumes showed similar patterns to the brain volume: all the cortical volumes decreased significantly with age. The insular volume only showed a marginal aging effect (*b* = −0.06; *p* = 0.0088), which was not significant after Bonferroni correction. All cortical volumes were different between the ethnic groups: Koreans had bigger cortical volumes than Caucasians (*b*’s <−0.3; *p*’s < 0.003). There were no significant sex differences after Bonferroni correction. Only weak sex differences were found in the parietal (*b* = −0.05; *p* = 0.021) and insular cortices (*b* = 0.07; *p* = 0.018).

### Subcortical Volumes and Socio-demographic Effects

Although other subcortical structures showed similar patterns to the cortical structures, some subcortical volumes appeared out of the ordinary pattern. The pallidus was not significantly affected by age (*b* = 0.04; *p* > 0.1). In contrast to all cortical structures that were bigger in Koreans, the ventricle (*b* = 0.08; *p* < 0.003) and caudate (*b* = 0.09; *p* < 0.003) were bigger in Caucasians, and the putamen displayed no ethnic difference (*b* = −0.01; *p* > 0.7). There were significant sex differences in two regions: the caudate was bigger in women (*b* = −0.12; *p* < 0.003) and the amygdala was bigger in men (*b* = 0.12; *p* < 0.003). Only a weak education effect was found in the accumbens (*b* = 0.07; *p* = 0.045).

### Aging Slopes in Koreans and Caucasians

**Figure 1:**
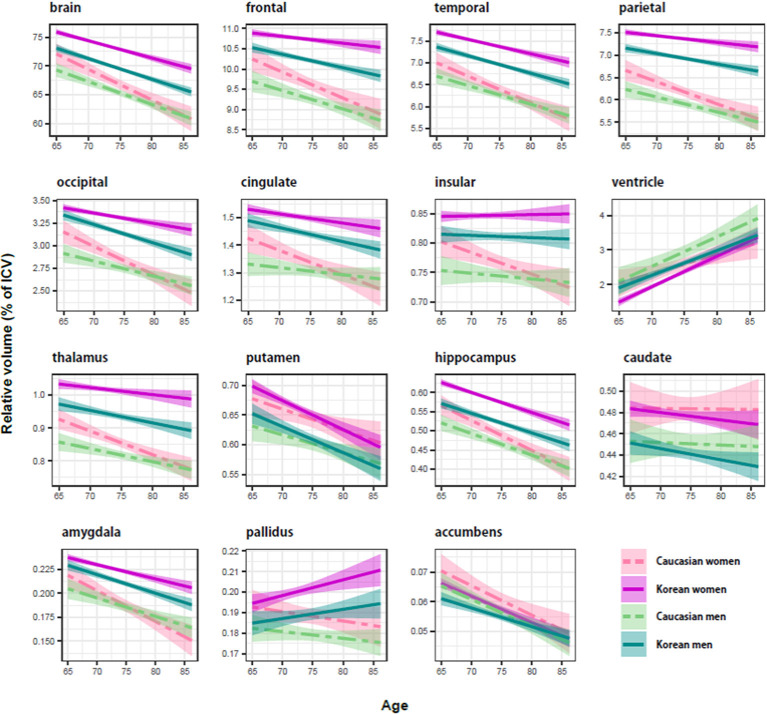
Ethnicity and sex differences of the aging brain in relative size (percentage of intracranial volume). The shaded region around each trajectory shows ± 1 SE of the mean. In cortical structures, Koreans (solid line) seemed to be bigger than Caucasians (dashed line), and women (red line) appeared to be bigger than men (cyan line). Compared with the cortical structure, there were no consistent effects of ethnicity and sex on subcortical structures.

The results of the previous regression analysis could be simply summarized: the brain structures were affected by age, ethnicity, and sex, not by education. Therefore, we classified the subjects into four groups based on ethnicity and sex: female and male groups of Koreans and of Caucasians, and determined the aging effect on the brain structures of each group, using a regression analysis with age as independent variables (Figure 1). As a dependent variable, a volume relative to intracranial volume (ICV) was used because age and ICV were significantly correlated (*r* = 0.12; *p* < 0.003). Bootstrap technique was used for producing the CI of an aging slope or a beta coefficient of age (Tables s 3, 4).

**Table 3:** Ethnicity and sex differences in aging slopes of cortical and subcortical structures.

	Female				Male			
	Caucasian		Korean		Caucasian		Korean	
	Slope	95% CI	Slope	95% CI	Slope	95% CI	Slope	95% CI
Brain volume	**−0.791**	[−1.152, −0.574]	**−0.408**	[−0.486, −0.329]	**−0.612**	[−0.760, −0.457]	**−0.513**	[−0.598, −0.428]
Cortical volumes	
Frontal	**−0.664**	[−1.018, −0.342]	**−0.155**	[−0.257, −0.050]	**−0.491**	[−0.741, −0.245]	**−0.323**	[−0.422, −0.221]
Temporal	**−0.941**	[−1.268, −0.632]	**−0.439**	[−0.544, −0.338]	**−0.684**	[−0.955, −0.433]	**−0.570**	[−0.691, −0.456]
Parietal	**−0.828**	[−1.141, −0.517]	**−0.207**	[−0.315, −0.098]	**−0.584**	[−0.848, −0.315]	**−0.349**	[−0.473, −0.222]
Occipital	**−1.114**	[−1.541, −0.722]	**−0.344**	[−0.480, −0.205]	**−0.615**	[−0.903, −0.309]	**−0.659**	[−0.820, −0.496]
Cingulate	**−0.649**	[−1.069, −0.275]	**−0.218**	[−0.364, −0.074]	−0.196	[−0.460, 0.059]	**−0.347**	[−0.515, −0.182]
Insular	**−0.478**	[−0.835, −0.145]	0.025	[−0.105, 0.162]	−0.130	[−0.448, 0.177]	−0.048	[−0.203, 0.110]
Subcortical volumes	
Ventricle	**2.187**	[0.549, 3.741]	**4.176**	[3.527, 4.815]	**2.869**	[1.935, 3.904]	**2.796**	[2.181, 3.427]
Thalamus	**−0.832**	[−1.286, −0.534]	**−0.210**	[−0.381, −0.023]	**−0.490**	[−0.774, −0.207]	**−0.406**	[−0.605, −0.205]
Putamen	−0.550	[−1.058, 0.008]	**−0.734**	[−0.960, −0.500]	**−0.496**	[−0.835, −0.136]	**−0.714**	[−0.976, −0.441]
Hippocampus	**−1.588**	[−2.074, −1.122]	**−0.886**	[−1.057, −0.723]	**−1.220**	[−1.605, −0.825]	**−0.977**	[−1.193, −0.761]
Caudate	−0.010	[−0.520, 0.571]	−0.144	[−0.322, 0.036]	−0.058	[−0.433, 0.351]	−0.236	[−0.476, 0.003]
Amygdala	**−1.697**	[−2.375, −1.078]	**−0.652**	[−0.835, −0.468]	**−1.046**	[−1.558, −0.531]	**−0.918**	[−1.159, −0.683]
Pallidus	−0.243	[−0.664, 0.187]	**0.383**	[0.140, 0.613]	−0.183	[−0.493, 0.149]	0.236	[−0.089, 0.575]
Accumbens	**−1.635**	[−2.409, −0.759]	**−1.465**	[−1.784, −1.147]	**−1.602**	[−2.345, −0.932]	**−1.137**	[−1.502, −0.772]

*Values are presented in % per year, and based on 10,000 iterations of linear regression analysis. Slope is presented in bold if the 95% confidence interval (CI) does not include the value of zero*.

**Table 4:** *APOE* ε4 effects on aging slopes of cortical and subcortical structures in women.

	Female Caucasian				Female Korean			
	*APOE* ε4 non-carrier		*APOE* ε4 carrier		*APOE* ε4 non-carrier		*APOE* ε4 carrier	
	Slope	95% CI	Slope	95% CI	Slope	95% CI	Slope	95% CI
Brain volume	**−0.624**	[−0.876, −0.405]	**−1.234**	[−2.39, −0.739]	**−0.387**	[−0.481, −0.290]	**−0.573**	[−0.771, −0.376]
Cortical volumes	
Frontal	**−0.495**	[−0.859, −0.163]	**−1.078**	[−2.008, −0.296]	−0.104	[−0.230, 0.034]	−0.264	[−0.555, −0.006]
Temporal	**−0.713**	[−1.043, −0.385]	**−1.446**	[−2.199, −0.708]	**−0.451**	[−0.582, −0.326]	**−0.381**	[−0.632, −0.101]
Parietal	**−0.635**	[−0.994, −0.281]	**−1.370**	[−2.029, −0.704]	**−0.203**	[−0.334, −0.064]	−0.270	[−0.598, 0.057]
Occipital	**−0.862**	[−1.347, −0.420]	**−1.830**	[−2.599, −0.965]	**−0.310**	[−0.474, −0.136]	**−0.512**	[−0.870, −0.129]
Cingulate	**−0.528**	[−0.963, −0.124]	**−0.960**	[−1.964, −0.153]	−0.146	[−0.317, 0.028]	−0.261	[−0.637, 0.094]
Insular	−0.225	[−0.563, 0.116]	**−1.135**	[−2.111, −0.325]	0.005	[−0.161, 0.176]	0.277	[−0.054, 0.594]
Subcortical volumes	
Ventricle	1.694	[−0.152, 3.487]	**3.116**	[0.103, 5.803]	**4.183**	[3.472, 4.929]	**4.360**	[2.144, 6.486]
Thalamus	**−0.665**	[−0.994, −0.359]	**−1.261**	[−2.637, −0.487]	−0.213	[−0.416, 0.000]	−0.465	[−0.959, 0.076]
Putamen	−0.277	[−0.859, 0.371]	**−1.693**	[−2.473, −0.730]	**−0.741**	[−0.995, −0.483]	−0.602	[−1.248, 0.052]
Hippocampus	**−1.383**	[−1.906, −0.880]	**−2.104**	[−3.304, −1.037]	**−0.967**	[−1.163, −0.753]	**−0.636**	[−1.055, −0.227]
Caudate	0.235	[−0.327, 0.954]	**−0.942**	[−2.032, −0.111]	−0.055	[−0.271, 0.169]	−0.288	[−0.777, 0.140]
Amygdala	**−1.394**	[−2.098, −0.700]	**−2.419**	[−4.208, −0.973]	**−0.561**	[−0.754, −0.367]	**−0.700**	[−1.110, −0.145]
Pallidus	0.041	[−0.458, 0.512]	**−1.263**	[−1.965, −0.599]	**0.379**	[0.094, 0.662]	0.343	[−0.381, 0.998]
Accumbens	**−1.092**	[−2.029, −0.053]	**−2.899**	[−4.242, −1.525]	**−1.515**	[−1.906, −1.111]	**−1.364**	[−2.178, −0.576]

*Values are presented in % per year, and based on 10,000 iterations of linear regression analysis. Slope is presented in bold if the 95% confidence interval (CI) does not include the value of zero*.

**Figure 2:**
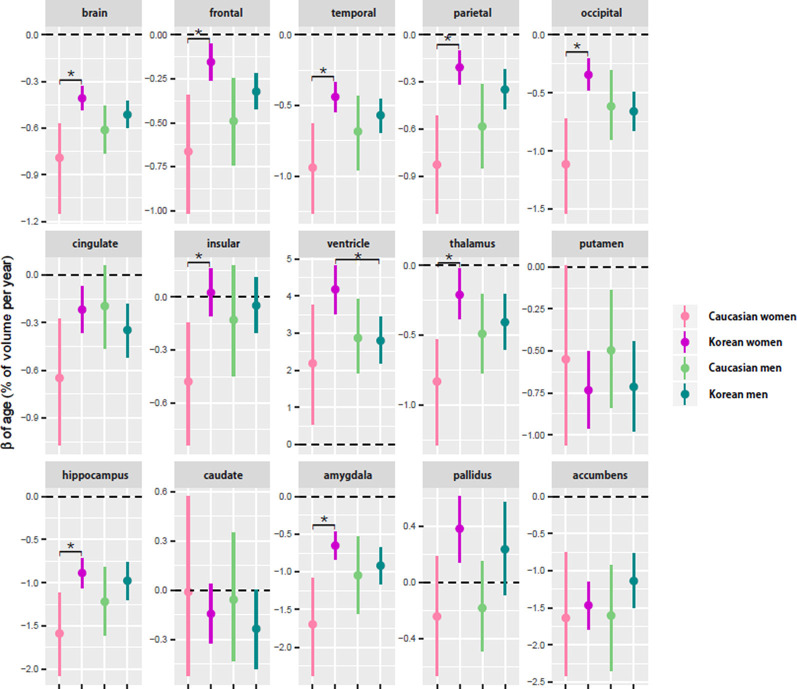
Comparison of aging slopes of brain structures between Caucasians and Koreans. The aging slopes were compared among the four groups: Caucasian men and women and Korean men and women. Ethnic differences in aging slope were found between the female groups. The aging slopes and the confidence intervals (CIs) were estimated based on 10,000 bootstrap iterations of linear regression analysis using each regional volume relative to intracranial volume (ICV) as a dependent variable. Asterisk denotes non-overlap of two CIs or statistical significance.

Regardless of ethnicity or sex, the brain shrank with age (Table 3). Although the aging slopes of brain volume were statistically significant in all the groups, a significant group difference of the aging slopes was found in a comparison of the relative volumes between the two female ethnic groups. In female aging slope, Caucasians were significantly steeper than Koreans (Figure 2).

Four of the six cortical structures, the frontal, temporal, parietal, and occipital cortices, showed the same pattern as the brain volume did: the four cortical volumes decayed during aging in all the groups (Table 3) and confirmed that Caucasian women had steeper aging slopes than Korean women. The cingulate cortex appeared to follow the main trend in general, although the ethnic difference in women was not statistically significant. Interestingly, the insula largely did not seem to be affected by aging except that it had a significant slope in female Caucasians.

Among the eight subcortical structures, four, the thalamus, hippocampus, amygdala, and accumbens, followed the general trends shown above. The four subcortical volumes decreased during aging for all the groups and kept the tendency of the steeper slopes in Caucasian women than in Korean women, although the CIs of the female groups were overlapped in one of the four structures, the accumbens. However, the other subcortical structures showed two other types of patterns besides the general trend. The first type is influenced by aging as well, but with a weak reverse ethnic difference. The ventricle expanded with age for all the groups and showed a weak reverse ethnic difference: Korean women had a steeper albeit insignificant slope than Caucasian women. The putamen decreased with age for all the groups except Caucasian women whose CI spanned a little over zero, and also showed weak reverse ethnic differences, such as the ventricle. The second type did not appear to be affected by aging. The caudate and pallidus were relatively stable during aging in all the groups and in all other groups than Korean women, respectively.

### Aging Slopes in Women: *APOE* ε4 Carriers vs. Non-carriers

**Figure 3:**
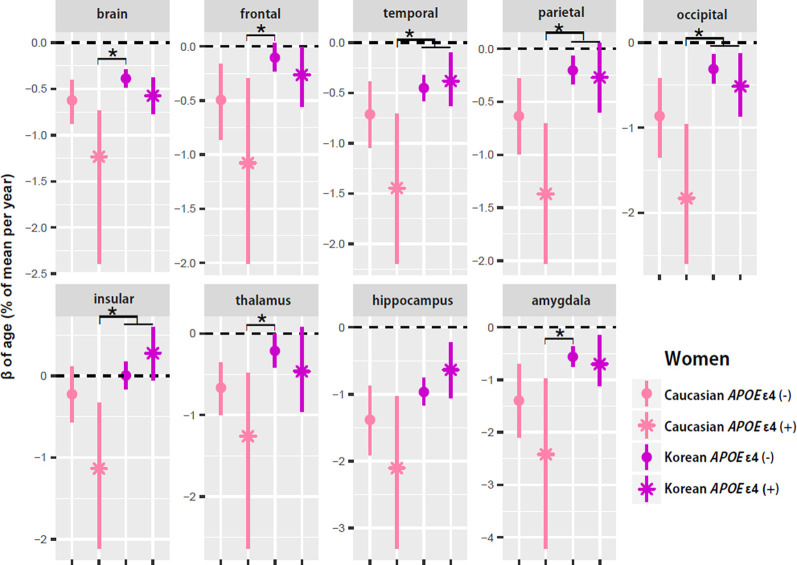
*APOE* ε4 effects on the aging slopes of women. The pairs showing group differences were Caucasian *APOE* ε4 carriers vs. Korean non-carriers or carriers. The aging slopes of females were compared among the four groups: Caucasian *APOE* ε4 carriers and non-carriers and Korean *APOE* ε4 carriers and non-carriers. The aging slopes and the confidence intervals (CIs) were estimated based on 10,000 bootstrap iterations of linear regression analysis using each regional volume relative to ICV as a dependent variable. Asterisk denotes non-overlap of two CIs or statistical significance.

To test whether ethnic differences in women were due to *APOE* ε4, the two female groups were divided according to *APOE* ε4 carrier status, and their aging slopes were compared (Figure 3). The results were consistent with the hypothesis. The aging slopes of Caucasian *APOE* ε4 carriers were significantly steeper than those of Korean *APOE* ε4 non-carriers or carriers in most of the brain structures showing the ethnic differences in women. It explains much of the ethnic difference in females.

### *APOE* ε4 Effects on Cortical and Subcortical Structures in Aging Slope

**Figure 4:**
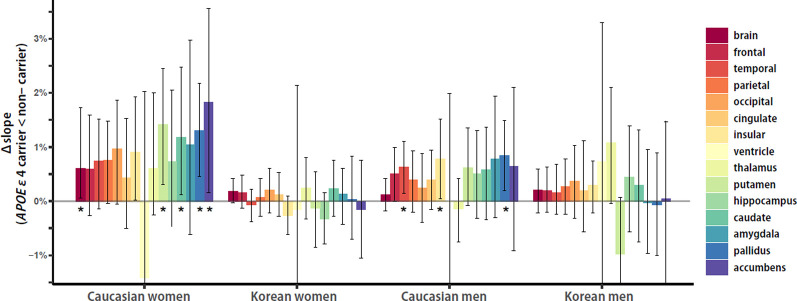
Ethnicity and sex differences in *APOE* ε4 effects on aging slopes. Overall, Caucasian women seemed to be the biggest in the *APOE* ε4 effects, and Caucasian men seemed to be the second. Caucasian women showed five significant effects in the brain and the four subcortical structures, such as the putamen, caudate, pallidus, and accumbens, whereas Caucasian men had three significant effects in the two cortical structures and one subcortical structure, such as the temporal and insular cortices and pallidus. Korean women and men did not have any significant effect. The *APOE* ε4 effects of each group were calculated as the differences of aging slopes between *APOE* ε4 carriers and non-carriers. The aging slopes, the differences, and the confidence intervals were estimated based on 10,000 bootstrap iterations of linear regression analysis using each regional volume relative to ICV as a dependent variable. Asterisk denotes statistical significance.

To investigate the within-group *APOE* ε4 effects on the aging slopes of cortical and subcortical structures, the effect sizes of *APOE* ε4, or the distance from the aging slope of *APOE* ε4 non-carriers to the aging slope of carriers, were calculated and compared between ethnicity and sex groups. Overall, Caucasian women seemed to be the biggest in the *APOE* ε4 effects, and Caucasian men seemed to be the second. Caucasian women showed five significant effects in the brain and the four subcortical structures, such as the putamen, caudate, pallidus, and accumbens, whereas Caucasian men had three significant effects in the two cortical and one subcortical structures, such as the temporal and insular cortices and pallidus (Figure 4). Korean women and men did not have any significant effect. The results support that the effects of the genetic risk factor may vary with ethnicity and sex.

### Norms of Brain Structures of Koreans and Caucasians

The previous analysis using bootstrap technique revealed that aging effects on some brain structures were divergent across ethnicity and sex. Therefore, the norms of the brain structures need to be subdivided according to ethnicity, sex, and age group of a 5-year range. The norms were evaluated in relative volume to ICV for Koreans (Supplementary Table S1) and Caucasians (Supplementary Table S2). Additionally, the norms of absolute volume were assessed for Koreans (Supplementary Table S3) and Caucasians (Supplementary Table S4).

## Discussion

To our knowledge, this is the first study to investigate ethnical disparities in normal aging by using a large single-scanner sample of old people with a narrow age range, which is supportive to make robust conclusions in the absence of differing study methodologies (Shen et al., 2017). Although Caucasian brain images were much different from our images in terms of MRI protocols, scanner models, image resolution, and so on, the volume loss per year rate conceptually very similar to the aging slope is revealed to be consistent across the different cohorts and independent from the scanner applied (Schippling et al., 2017). Our results from this unusually large, single-scanner sample provide one of the most extensive characterizations of age-associated decay rate in the human brain.

Age and ethnicity were found to significantly affect the brain volume. The cortical and insular volumes revealed a gradual decrease by age and a marginal aging effect, respectively. In addition, all cortical structures were significantly bigger in Koreans than in Caucasians, except for the ventricle and caudate that were significantly bigger in Caucasians, and the putamen showed no significant difference. Moreover, sex disparity was observed in the caudate and amygdala, which were significantly bigger in women and in men, respectively. Notably, the aging slope for Caucasian women was significantly steeper than that for Korean women. According to our data, it seemed to be associated with the extent of decay during aging in four cortical structures, including the frontal, temporal, parietal, and occipital cortices, that revealed steeper aging slope in female Caucasians than in female Koreans. In addition, the insula significantly decreased age dependently in Caucasian women only.

The prevalence of *APOE* ε4 needs to be considered when interpreting our results that the proportion of *APOE* ε4 carriers was previously reported to be higher in Caucasians (Shi et al., 2017; Sepulcre et al., 2018). The susceptibility for *APOE* ε4 effects was highest in Caucasians, followed by Koreans, and direct comparison among *APOE* ε4 carriers indicated significantly steeper aging slopes in Caucasians than in Koreans. These results suggest that the ethnicity may be independently associated with the aging slope, and furthermore that the ethnicity is a decisive factor for the extent of brain aging along with some known risk factors, such as *APOE* ε4, potentially due to genetic disparities.

Our results were consistent with previous studies of sex differences in *APOE* ε4 effects. In the normal populations, 20–30% of Caucasian individuals carry *APOE* ε4, whereas 17% of East Asians do (Saunders et al., 1993; Farrer et al., 1997). Our results showed similar proportions of *APOE* ε4 carriers. Women with *APOE* ε4 genotype are known to have an increased risk of Alzheimer’s disease (AD) compared with men with *APOE* ε4. Farrer et al. (1997) and Neu et al. (2017) reported the increased risk of Caucasian women with *APOE* ε4 compared with Caucasian men between the ages of 65 and 75 years. We also found that *APOE* ε4 made steeper aging slopes of the brain structures in Caucasian women. Previous studies explained that the increased risk of Caucasian women with *APOE* ε4 is attributed to female sex hormones, such as estrogen (Yaffe, 2001; Jacobs et al., 2013). Additionally, there are some evidence of sex differences in the association between *APOE* ε4 carrier status and AD biomarkers (Farrer et al., 1997; Raber et al., 1998; Damoiseaux et al., 2012). In Koreans, we found that *APOE* ε4 has no difference in AD risk between female and male carriers, but only the increased aging slope of the ventricle in women regardless of *APOE* ε4 carrier status (Figure 2).

The brain morphometry of East Asians has been known to be different from that of Caucasians and Africans although AD-associated patterns of brain atrophy are reported to exhibit no ethnic difference (Fan et al., 2019). East Asian brains are the largest in terms of cranial size (Beals et al., 1984; Howells, 1990; Rushton, 2000). East Asian hemispheres are shorter and wider than Caucasian hemispheres using MRI (Zilles et al., 2001). East Asians are found to have larger temporal and cingulate gyri than Caucasians using a surface-based morphometry method (Chee et al., 2011; Tang et al., 2018). In addition, Africans were reported to exhibit larger left orbito-frontal cortex than Caucasians (Isamah et al., 2010). Furthermore, the authors stressed the inclusion of ethnic minorities in clinical research because the inclusion is critical for generalizability of research findings. Even in this context, the normative information stratified by ethnicity is important. We also presented the normative information on volume estimates of the brain structures of the elderly Korean people in the subdivided age groups. This normative information of the aging brain stratified by ethnicity provides the age-related reference ranges quantified to replace visual assessment that is dependent on the subjective judgment of the examiner and needed to facilitate research and precise clinical decision-making close to personalized diagnosis of neurodegenerative diseases.

In conclusion, our results provide that gender and ethnicity contribute differentially to the global brain atrophy observed in normal elderly Korean and Caucasian males and females, and discover that changes in brain volume may be due to the ethnicity-dependent effects of the genetic risk of carrying *APOE* ε4.

## Limitation

The Caucasian sample was not a single-scanner dataset, but MRI images from diverse scanner models including our model. The scanner diverseness may increase the error variance of the results in Caucasians, but just was expected to weaken the statistical significance of the ethnic differences in our results. This study is a cross-sectional study that has been under attack because it might be inappropriate for individual variability in age-related change (Raz and Lindenberger, 2011). However, longitudinal MRI studies also often have had limitations as relatively small numbers of subjects, short overall periods of follow-up, and/or small number of follow-up assessments (Salthouse, 2011; Schuster et al., 2015). The cross-sectional MRI studies have consistently revealed that increased age is associated with brain shrinkage, and have been supported by most of the longitudinal studies (Fjell et al., 2009a).

## Data Availability Statement

Publicly available datasets were analyzed in this study. This data can be found here: Alzheimer’s Disease Neuroimaging Initiative database^1^.

## Ethics Statement

The studies involving human participants were reviewed and approved by the Institutional Review Board of Chosun University Hospital, South Korea. The patients/participants provided their written informed consent to participate in this study.

## Author Contributions

YC conducted the imaging analysis and contributed to the preliminary draft writing. JL and KC conducted the statistical analysis. ES and IC interpreted the neuropsychological results. HK, M-KS, S-MC, SC, and BK interviewed and examined the subjects and reviewed the brain MRIs. BK and KL designed the study, wrote the manuscript, and provided the overall supervision for the project.

## Conflict of Interest

The authors declare that the research was conducted in the absence of any commercial or financial relationships that could be construed as a potential conflict of interest.

## Footnotes

^1^http://adni.loni.usc.edu

^2^http://adni.loni.usc.edu/methods/documents/mri-protocols/

^3^http://surfer.nmr.mgh.harvard.edu/

^4^https://www.r-project.org/

## Supplementary Material

FIGURE S1 Ethnicity and sex effects on the aging brain in absolute size. The shaded region around each trajectory shows ±1 SE of the mean. Compared to Figure 1, sex differences became reverse and apparent, especially in Caucasians: men were bigger than women in all the brain regions.

FIGURE S2 Aging slope in absolute size. The caudate produced different results between absolute and relative volumes. In absolute size, the caudate seemed to have ethnic differences: Koreans had significant slopes, but Caucasians did not. In relative size, the caudate had largely insignificant slopes, and a significant but marginal slope of which CI was close to 0. Values are presented in cm^3^ per decade.

TABLE S1 Percentage volume ratios of cortical and subcortical structure to ICV in the Korean elderly.

TABLE S2 Percentage volume ratios of cortical and subcortical structure to ICV in the Caucasian elderly.

TABLE S3 Volumes of cortical and subcortical structures in the Korean elderly.

TABLE S4 Volumes of cortical and subcortical structures in the Caucasian elderly.

TABLE S5 Differences of aging slopes between *APOE* ε4 carriers and non-carriers.

## References

[B100] Beals K. L. Smith C. L. Dodd S. M. (1984). Brain size, cranial morphology, climate, and time machines. Curr. Anthropol. 25, 301–330. 10.1086/203138

[B1] Bonomini F. Filippini F. Hayek T. Aviram M. Keidar S. Rodella L. F.. (2010). Apolipoprotein E and its role in aging and survival. Exp. Gerontol. 45, 149–157. 10.1016/j.exger.2009.11.006 19941948

[B101] Chee M. W. Zheng H. Goh J. O. Park O. Sutton B. P. (2011). Brain structure in young and old East asians and Westerners: comparisons of structural volume and cortical thickness. J. Cogn. Neurosci. 23, 1065–1079. 10.1162/jocn.2010.21513 20433238 PMC3361742

[B2] Dale A. M. Fischl B. Sereno M. I. (1999). Cortical surface-based analysis. I. segmentation and surface reconstruction. NeuroImage 9, 179–194. 10.1006/nimg.1998.0395 9931268

[B3] Damoiseaux J. S. Seeley W. W. Zhou J. Shirer W. R. Coppola G. Karydas A.. (2012). Gender modulates the APOE ε4 effect in healthy older adults: convergent evidence from functional brain connectivity and spinal fluid tau levels. J. Neurosci. 32, 8254–8262. 10.1523/JNEUROSCI.0305-12.2012 22699906 PMC3394933

[B4] Davison A. C. Hinkley D. V. (1997). Bootstrap Methods and Their Application. Cambridge: Cambridge University Press.

[B5] Desikan R. S. Ségonne F. Fischl B. Quinn B. T. Dickerson B. C. Blacker D.. (2006). An automated labeling system for subdividing the human cerebral cortex on MRI scans into gyral based regions of interest. NeuroImage 31, 968–980. 10.1016/j.NeuroImage.2006.01.021 16530430

[B7] Fan J. Tse M. Carr J. S. Miller B. L. Kramer J. H. Rosen H. J.. (2019). Alzheimer disease-associated cortical atrophy does not differ between chinese and whites. Alzheimer Dis. Assoc. Disord. 33, 186–193. 10.1097/wad.0000000000000315 31094707 PMC6527333

[B8] Farrer L. A. Cupples L. A. Haines J. L. Hyman B. Kukull W. A. Mayeux R.. (1997). Effects of age, sex and ethnicity on the association between apolipoprotein E genotype and Alzheimer disease. A meta-analysis. APOE and Alzheimer disease meta analysis consortium. JAMA 278, 1349–1356. 9343467

[B9] Fischl B. Dale A. M. (2000). Measuring the thickness of the human cerebral cortex from magnetic resonance images. Proc. Natl. Acad. Sci. U S A. 97, 11050–11055. 10.1073/pnas.200033797 10984517 PMC27146

[B10] Fischl B. Liu A. Dale A. M. (2001). Automated manifold surgery: constructing geometrically accurate and topologically correct models of the human cerebral cortex. IEEE Trans. Med. Imaging 20, 70–80. 10.1109/42.906426 11293693

[B11] Fischl B. Salat D. H. Busa E. Albert M. Dieterich M. Haselgrove C.. (2002). Whole brain segmentation: automated labeling of neuroanatomical structures in the human brain. Neuron 33, 341–355. 10.1016/s0896-6273(02)00569-x 11832223

[B12] Fischl B. Sereno M. I. Dale A. (1999a). Cortical surface-based analysis: ii: inflation, flattening and a surface-based coordinate system. NeuroImage 9, 195–207. 10.1006/nimg.1998.0396 9931269

[B13] Fischl B. Sereno M. I. Tootell R. B. Dale A. M. (1999b). High-resolution intersubject averaging and a coordinate system for the cortical surface. Hum. Brain Mapp. 8, 272–284. 10.1002/(sici)1097-0193(1999)8:4<272::aid-hbm10>3.0.co;2-4 10619420 PMC6873338

[B14] Fischl B. Van Der Kouwe A. Destrieux C. Halgren E. Ségonne F. Salat D. H.. (2004). Automatically parcellating the human cerebral cortex. Cereb. Cortex 14, 11–22. 10.1093/cercor/bhg087 14654453

[B16] Fjell A. M. Walhovd K. B. Fennema- Notestine C. Mcevoy L. K. Hagler D. J. Holland D.. (2009a). One-year brain atrophy evident in healthy aging. J. Neurosci. 29, 15223–15231. 10.1523/jneurosci.3252-09.2009 19955375 PMC2827793

[B17] Fjell A. M. Westlye L. T. Amlien I. Espeseth T. Reinvang I. Raz N.. (2009b). High consistency of regional cortical thinning in aging across multiple samples. Cereb. Cortex 19, 2001–2012. 10.1093/cercor/bhn232 19150922 PMC2733683

[B18] Folstein M. F. Folstein S. E. Mchugh P. R. (1975). “Mini-mental state.” A practical method for grading the cognitive state of patients for the clinician. J. Psychiatr. Res. 12, 189–198. 10.1016/0022-3956(75)90026-6 1202204

[B19] Fox N. C. Schott J. M. (2004). Imaging cerebral atrophy: normal ageing to Alzheimer’s disease. Lancet 363, 392–394. 10.1016/s0140-6736(04)15441-x 15074306

[B20] He W. Goodkind D. Kowal P. (2016). “An Aging World: 2015.” (Washington, DC: U.S. Government Publishing Office).

[B102] Howells W. W. (1990). Skull shapes and the Map: Craniometric analyses in the dispersion of mordern homo. Cambridge: Peabody Museum Press.

[B21] Isamah N. Faison W. Payne M. E. MacFall J. Steffens D. C. Beyer J. L.. (2010). Variability in frontotemporal brain structure: the importance of recruitment of african americans in neuroscience research. PLoS One 5:e13642. 10.1371/journal.pone.0013642 21049028 PMC2964318

[B22] Jacobs E. G. Kroenke C. Lin J. Epel E. S. Kenna H. A. Blackburn E. H.. (2013). Accelerated cell aging in female APOE-ε4 carriers: implications for hormone therapy use. PLoS One 8:e54713. 10.1371/journal.pone.0054713 23418430 PMC3572118

[B23] Jee Y. H. Lee S. J. Jung K. J. Jee S. H. (2016). Alcohol intake and serum glucose levels from the perspective of a mendelian randomization design: the KCPS-II biobank. PLoS One 11:e0162930. 10.1371/journal.pone.0162930 27632197 PMC5025151

[B24] Kang Y. Jang S. Na D. L. (2012). Seoul Neuropsychological Screening Battery (SNSB). Seoul: Human Brain Research and Consulting Co.

[B25] Klein A. Tourville J. (2012). 101 labeled brain images and a consistent human cortical labeling protocol. Front. Neurosci. 6:171. 10.3389/fnins.2012.00171 23227001 PMC3514540

[B26] Ledig C. Schuh A. Guerrero R. Heckemann R. A. Rueckert D. (2018). Structural brain imaging in Alzheimer’s disease and mild cognitive impairment: biomarker analysis and shared morphometry database. Sci. Rep. 8:11258. 10.1038/s41598-018-29295-9 30050078 PMC6062561

[B27] Morris J. C. (1993). The clinical dementia rating (CDR): current version and scoring rules. Neurology 43, 2412–2414. 10.1212/wnl.43.11.2412-a 8232972

[B28] Neu S. C. Pa J. Kukull W. Beekly D. Kuzma A. Gangadharan P.. (2017). Apolipoprotein E genotype and sex risk factors for Alzheimer disease. JAMA Neurol. 74, 1178–1189. 10.1001/jamaneurol.2017.2188 28846757 PMC5759346

[B29] Raber J. Wong D. Buttini M. Orth M. Bellosta S. Pitas R. E.. (1998). Isoform-specific effects of human apolipoprotein E on brain function revealed in ApoE knockout mice: increased susceptibility of females. Proc. Natl. Acad. Sci. U S A. 95, 10914–10919. 10.1073/pnas.95.18.10914 9724804 PMC27995

[B30] Raz N. Lindenberger U. (2011). Only time will tell: cross-sectional studies offer no solution to the age-brain-cognition triangle: comment on Salthouse. Psychol. Bull. 137, 790–795. 10.1037/a0024503 21859179 PMC3160731

[B32] Reinvang I. Espeseth T. Westlye L. T. (2013). APOE-related biomarker profiles in non-pathological aging and early phases of Alzheimer’s disease. Neurosci. Biobehav. Rev. 37, 1322–1335. 10.1016/j.neubiorev.2013.05.006 23701948

[B33] Resnick S. M. Pham D. L. Kraut M. A. Zonderman A. B. Davatzikos C. (2003). Longitudinal magnetic resonance imaging studies of older adults: a shrinking brain. J. Neurosci. 23, 3295–3301. 10.1523/jneurosci.23-08-03295.2003 12716936 PMC6742337

[B103] Rushton J. P. (2000). Race, Evolution, and Behavior: A life history perspective. 3rd edn. Porto Huron: Charles Darwin Research Institute Press.

[B34] Salthouse T. A. (2011). Neuroanatomical substrates of age-related cognitive decline. Psychol. Bull. 137, 753–784. 10.1037/a0023262 21463028 PMC3132227

[B35] Saunders A. M. Strittmatter W. J. Schmechel D. George-Hyslop P. H. S. Pericak-Vance M. A. Joo S. H.. (1993). Association of apolipoprotein E allele ε4 with late-onset familial and sporadic Alzheimer’s disease. Neurology 43, 1467–1472. 10.1212/wnl.43.8.1467 8350998

[B36] Scahill R. I. Frost C. Jenkins R. Whitwell J. L. Rossor M. N. Fox N. C. (2003). A longitudinal study of brain volume changes in normal aging using serial registered magnetic resonance imaging. Arch. Neurol. 60, 989–994. 10.1001/archneur.60.7.989 12873856

[B37] Schippling S. Ostwaldt A.-C. Suppa P. Spies L. Manogaran P. Gocke C.. (2017). Global and regional annual brain volume loss rates in physiological aging. J. Neurol. 264, 520–528. 10.1007/s00415-016-8374-y 28054131

[B38] Schuster C. Elamin M. Hardiman O. Bede P. (2015). Presymptomatic and longitudinal neuroimaging in neurodegeneration-from snapshots to motion picture: a systematic review. J. Neurol. Neurosurg. Psychiatry. 86, 1089–1096. 10.1136/jnnp-2014-309888 25632156

[B39] Sepulcre J. Grothe M. J. D’oleire Uquillas F. Ortiz-Terán L. Diez I. Yang H.-S.. (2018). Neurogenetic contributions to amyloid β and τ spreading in the human cortex. Nat. Med. 24, 1910–1918. 10.1038/s41591-018-0206-4 30374196 PMC6518398

[B40] Shen X. Reus L. M. Cox S. R. Adams M. J. Liewald D. C. Bastin M. E.. (2017). Subcortical volume and white matter integrity abnormalities in major depressive disorder: findings from UK biobank imaging data. Sci. Rep. 7:5547. 10.1038/s41598-017-05507-6 28717197 PMC5514104

[B41] Shi Y. Yamada K. Liddelow S. A. Smith S. T. Zhao L. Luo W.. (2017). ApoE4 markedly exacerbates tau-mediated neurodegeneration in a mouse model of tauopathy. Nature 549, 523–527. 10.1038/nature24016 28959956 PMC5641217

[B42] Sled J. G. Zijdenbos A. P. Evans A. C. (1998). A nonparametric method for automatic correction of intensity nonuniformity in MRI data. IEEE Trans. Med. Imaging 17, 87–97. 10.1109/42.668698 9617910

[B43] Sowell E. R. Peterson B. S. Thompson P. M. Welcome S. E. Henkenius A. L. Toga A. W. (2003). Mapping cortical change across the human life span. Nat. Neurosci. 6, 309–315. 10.1038/nn1008 12548289

[B104] Tang Y. Zhao L. Lou Y. Shi Y. Fang R. Lin S.. (2018). Brain structure differences between Chinese and Caucasian cohorts: A comprehensive morphometry study. Hum. Brain Mapp. 39, 2147–2155. 10.1002/hbm.23994 29400417 PMC6625506

[B45] Weiner M. W. Veitch D. P. Aisen P. S. Beckett L. A. Cairns N. J. Cedarbaum J.. (2015). 2014 update of the Alzheimer’s disease neuroimaging initiative: a review of papers published since its inception. Alzheimers Dement. 11, e1–120. 10.1016/j.jalz.2014.11.001 26073027 PMC5469297

[B46] Yaffe K. (2001). Estrogens, selective estrogen receptor modulators and dementia: what is the evidence? Ann. N Y Acad. Sci. 949, 215–222. 10.1111/j.1749-6632.2001.tb04024.x 11795356

[B105] Zilles K. Kawashima R. Dabringhaus A. Fukuda H. Schormann T. (2001). Hemispheric shape of European and Japanese brains: 3-D MRI analysis of intersubject variability, ethnical, and gender differences. NeuroImage 13, 262–271. 10.1006/nimg.2000.0688 11162267

