# Corrigendum: The Aging Slopes of Brain Structures Vary by Ethnicity and Sex: Evidence From a Large Magnetic Resonance Imaging Dataset From a Single Scanner of Cognitively Healthy Elderly People in Korea

**DOI:** 10.3389/fnagi.2020.604238

**Published:** 2020-11-09

**Authors:** Yu Yong Choi, Jang Jae Lee, Kyu Yeong Choi, Eun Hyun Seo, IL Han Choo, Hoowon Kim, Min-Kyung Song, Seong-Min Choi, Soo Hyun Cho, Byeong C. Kim, Kun Ho Lee

**Affiliations:** ^1^Gwangju Alzheimer's Disease and Related Dementias (GARD) Cohort Research Center, Chosun University, Gwangju, South Korea; ^2^Biomedical Technology Center, Chosun University Hospital, Gwangju, South Korea; ^3^Department of Neuropsychiatry, Chosun University School of Medicine and Hospital, Gwangju, South Korea; ^4^Department of Neurology, Chosun University School of Medicine and Hospital, Gwangju, South Korea; ^5^Department of Neurology, Chonnam National University Medical School and Hospital, Gwangju, South Korea; ^6^Department of Biomedical Science, Chosun University, Gwangju, South Korea; ^7^Dementia Research Group, Korea Brain Research Institute, Daegu, South Korea

**Keywords:** normal aging, aging slope, norm, ethnic difference, sex difference

In the original article, we neglected to include the funder National Institutes of Health (NIH Grant U01 AG024904) and DOD ADNI (Department of Defense award number W81XWH-12-2-0012) to ADNI. The funding details of ADNI can be found at: http://adni.loni.usc.edu/about/funding/.

Alzheimer's Disease Neuroimaging Initiative (ADNI) was not included as an author in the published article. The corrected Statement appears below.

The ADNI project that provided Caucasian data was launched in 2003 as a public-private partnership, led by Principal Investigator Michael W. Weiner (see http://www.adni-info.org/ for up-to-date information).

The authors apologize for this error and state that this does not change the scientific conclusions of the article in any way. The original article has been updated.

