# Supplementary figures and images for: The Aging Slopes of Brain Structures Vary by Ethnicity and Sex: Evidence From a Large Magnetic Resonance Imaging Dataset From a Single Scanner of Cognitively Healthy Elderly People in Korea
Source: Front Aging Neurosci. 2020 Aug 12;12:233. doi: 10.3389/fnagi.2020.00233 (PMC7437271; doi:10.3389/fnagi.2020.00233)

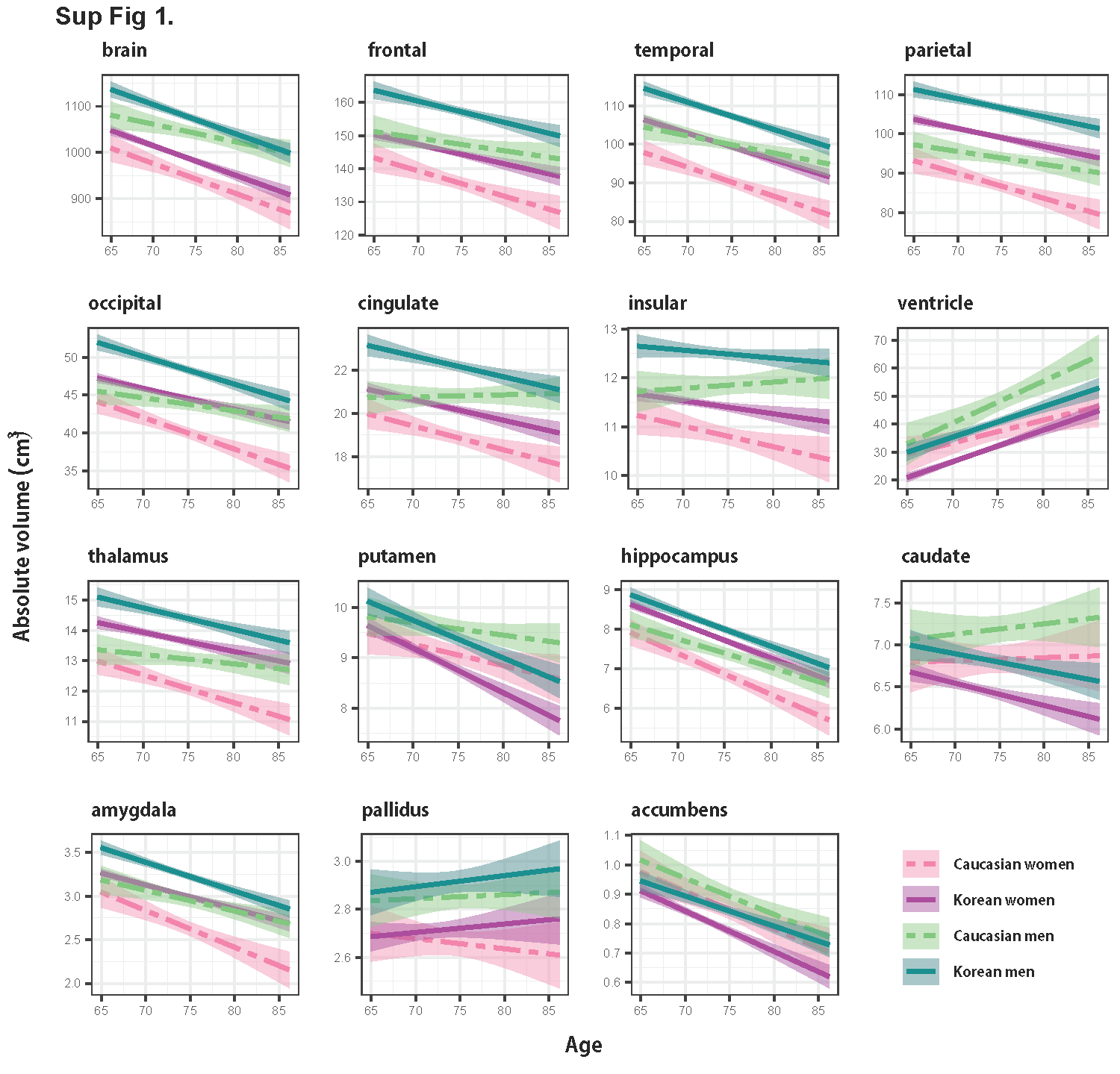

Supplement: FIGURE S1 — Ethnicity and sex effects on the aging brain in absolute size. The shaded region around each trajectory shows ±1 SE of the mean. Compared to Figure 1, sex differences became reverse and apparent, especially in Caucasians: men were bigger than women in all the brain regions. [file Image_1.TIFF]

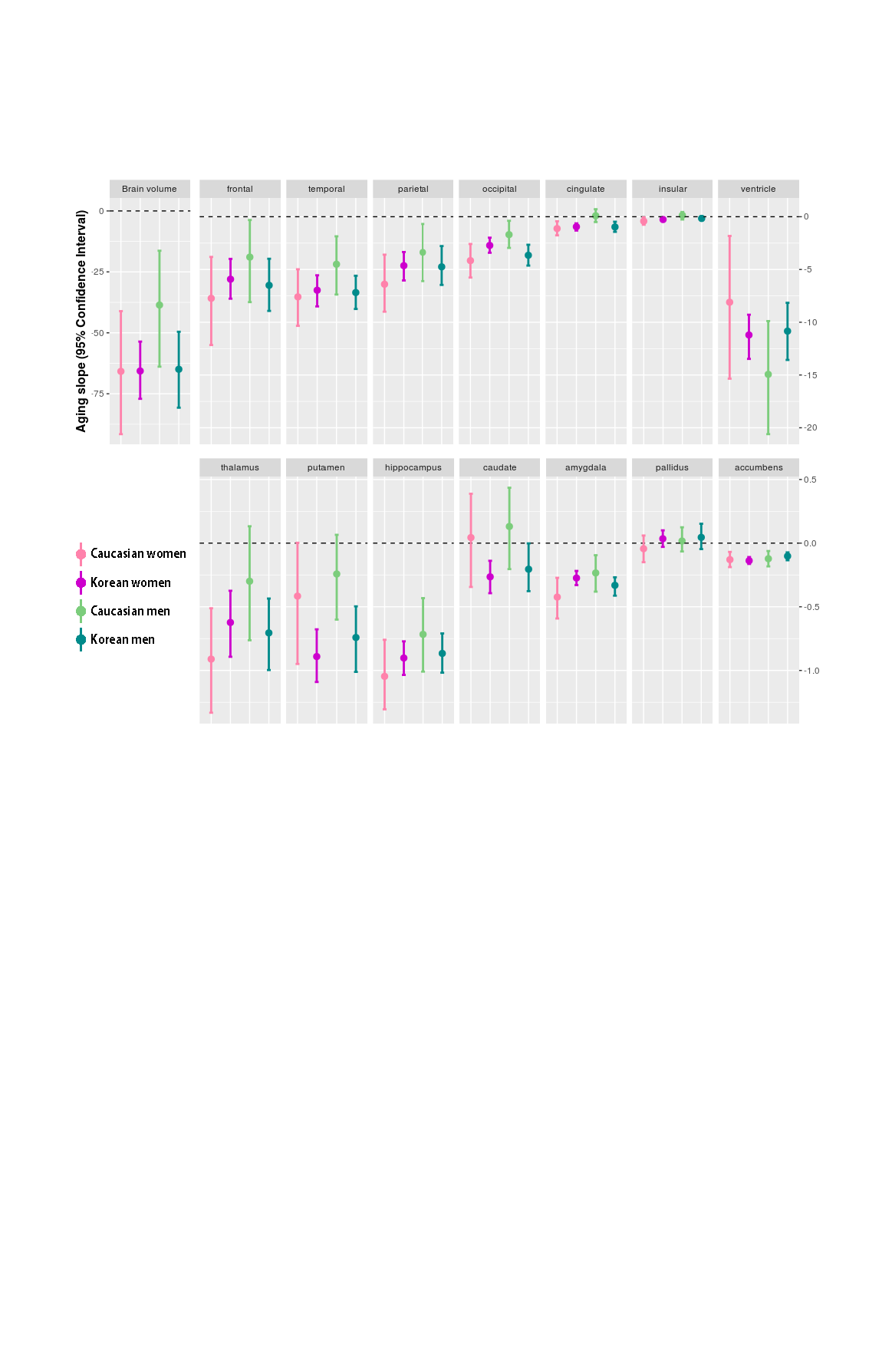

Supplement: FIGURE S2 — Aging slope in absolute size. The caudate produced different results between absolute and relative volumes. In absolute size, the caudate seemed to have ethnic differences: Koreans had significant slopes, but Caucasians did not. In relative size, the caudate had largely insignificant slopes, and a significant but marginal slope of which CI was close to 0. Values are presented in cm3 per decade. [file Image_2.TIF]
