# Supplementary material for: The Aging Slopes of Brain Structures Vary by Ethnicity and Sex: Evidence From a Large Magnetic Resonance Imaging Dataset From a Single Scanner of Cognitively Healthy Elderly People in Korea
Source: Front Aging Neurosci. 2020 Aug 12;12:233. doi: 10.3389/fnagi.2020.00233 (PMC7437271; doi:10.3389/fnagi.2020.00233)
Supplement: TABLE S1 — Percentage volume ratios of cortical and subcortical structure to ICV in the Korean elderly. [file Data_Sheet_1.pdf]

Supplementary Table 1. Percentage volume ratios of cortical and subcortical structure to ICV in the Korean elderly

	age group (female)								age group (male)							
	**65-69**		**70-75**		**75-79**		**80-84**		**65-69**		**70-75**		**75-79**		**80-84**	
	M	SD	M	SD	M	SD	M	SD	M	SD	M	SD	M	SD	M	SD
Brain volume	75.224	3.148	73.402	3.138*	72.267	3.213*	71.706	3.809	71.883	2.854	70.704	3.030*	68.550	2.856*	66.498	2.819*
Cortical volumes																
frontal	10.826	0.589	10.746	0.607	10.734	0.679	10.659	0.646	10.389	0.498	10.322	0.621	10.091	0.556*	9.969	0.478
temporal	7.618	0.441	7.456	0.458*	7.275	0.424*	7.299	0.477	7.214	0.361	7.135	0.462	6.820	0.417*	6.653	0.446*
parietal	7.465	0.461	7.380	0.442	7.332	0.481	7.337	0.413	7.062	0.418	7.012	0.426	6.839	0.456*	6.711	0.407
occipital	3.394	0.255	3.316	0.279*	3.301	0.260	3.272	0.266	3.264	0.258	3.211	0.271	3.085	0.274*	2.948	0.218*
cingulate	1.516	0.120	1.509	0.118	1.495	0.132	1.468	0.119	1.466	0.105	1.454	0.120	1.442	0.117	1.376	0.117*
insular	0.846	0.059	0.843	0.062	0.853	0.067	0.849	0.069	0.805	0.054	0.819	0.072	0.810	0.060	0.803	0.069
Subcortical volumes																
ventricle	1.723	0.611	2.157	0.731*	2.582	0.916*	2.882	0.859*	2.142	0.686	2.465	0.780*	2.663	0.809	3.328	0.900*
thalamus	1.035	0.099	1.005	0.094*	1.009	0.096	1.030	0.117	0.965	0.100	0.941	0.097	0.925	0.093	0.911	0.084
putamen	0.686	0.070	0.659	0.080*	0.644	0.088	0.625	0.093	0.630	0.081	0.624	0.074	0.605	0.081	0.566	0.083*
hippocampus	0.610	0.052	0.589	0.061*	0.563	0.058*	0.536	0.062*	0.556	0.057	0.537	0.060*	0.503	0.062*	0.484	0.053
caudate	0.482	0.046	0.476	0.053	0.476	0.050	0.479	0.049	0.442	0.051	0.448	0.051	0.438	0.048	0.432	0.051
amygdala	0.233	0.023	0.226	0.025*	0.220	0.025*	0.213	0.025	0.222	0.024	0.217	0.024	0.203	0.026*	0.195	0.024
pallidus	0.198	0.027	0.198	0.033	0.205	0.029*	0.212	0.022	0.186	0.029	0.189	0.022	0.190	0.030	0.191	0.029
accumbens	0.064	0.012	0.059	0.010*	0.056	0.010*	0.051	0.009*	0.058	0.009	0.057	0.010	0.054	0.011*	0.049	0.010*
n	233		239		132		34		83		128		106		53	
age (y)	67.7	1.4	72.5	1.4	76.8	1.3	81.9	1.5	67.9	1.3	72.5	1.4	77.3	1.4	82.5	1.7
MMSE	27.6	1.8	27.1	2.1	26.6	2.4	25.1	2.9	28.4	1.2	28.0	1.6	27.6	1.6	27.0	1.6
Education (y)	9.4	4.0	8.5	3.8	7.2	4.0	5.8	4.1	13.0	4.1	12.3	4.0	11.9	4.0	11.6	3.7

Volumes are presented in %.

* indicates statistical significance of comparision with the immediately preceding age group ($p < 0.05$) using pairwise t tests whose p values were adjusted by the Benjamini-Hochberg method.

MMSE, mini mental state examination; y, year.

Supplementary Table 2. Percentage volume ratios of cortical and subcortical structure to ICV in the Caucasian elderly

	age group (female)								age group (male)							
	**65-69**		**70-75**		**75-79**		**80-84**		**65-69**		**70-75**		**75-79**		**80-84**	
	M	SD	M	SD	M	SD	M	SD	M	SD	M	SD	M	SD	M	SD
Brain volume	72.458	6.277	67.400	5.277*	65.889	5.130	62.964	3.412*	68.791	3.921	66.655	3.303*	63.689	3.813*	62.213	3.249
Cortical volumes																
frontal	10.282	0.948	9.643	0.878*	9.568	0.987	9.109	0.858	9.763	0.982	9.358	0.768	9.024	0.757*	8.952	0.762
temporal	7.104	0.681	6.403	0.706*	6.376	0.678	5.908	0.475*	6.778	0.651	6.358	0.512*	6.095	0.512*	5.984	0.540
parietal	6.756	0.523	6.153	0.666*	6.103	0.757	5.799	0.515	6.307	0.661	5.982	0.488*	5.662	0.549*	5.741	0.574
occipital	3.206	0.294	2.860	0.360*	2.785	0.374	2.622	0.356	3.025	0.342	2.769	0.300*	2.662	0.288	2.656	0.292
cingulate	1.437	0.145	1.339	0.143*	1.339	0.156	1.264	0.155	1.339	0.127	1.322	0.118	1.262	0.144	1.311	0.116
insular	0.814	0.102	0.763	0.067*	0.769	0.079	0.727	0.063*	0.795	0.092	0.736	0.068*	0.730	0.064	0.750	0.076
Subcortical volumes																
ventricle	2.036	1.122	2.428	1.175	2.726	0.974	3.136	1.231	2.116	0.669	2.592	0.980	3.462	1.541*	3.575	1.205
thalamus	0.956	0.106	0.851	0.080*	0.848	0.083	0.805	0.064*	0.853	0.093	0.835	0.088	0.794	0.065*	0.790	0.074
putamen	0.708	0.109	0.639	0.073*	0.621	0.082	0.646	0.102	0.657	0.079	0.602	0.075*	0.587	0.075	0.588	0.074
hippocampus	0.565	0.061	0.499	0.086*	0.480	0.070	0.422	0.069*	0.519	0.062	0.486	0.063	0.427	0.059*	0.433	0.061
caudate	0.506	0.076	0.474	0.064	0.481	0.063	0.492	0.086	0.451	0.049	0.452	0.056	0.449	0.061	0.448	0.071
amygdala	0.227	0.035	0.185	0.039*	0.184	0.041	0.167	0.035	0.215	0.029	0.188	0.030*	0.174	0.030*	0.177	0.034
pallidus	0.191	0.025	0.191	0.026	0.184	0.020	0.186	0.025	0.176	0.018	0.182	0.020	0.179	0.020	0.174	0.019
accumbens	0.074	0.014	0.060	0.018*	0.059	0.016	0.055	0.013	0.072	0.016	0.056	0.012*	0.053	0.013	0.052	0.014
n	23		65		51		24		16		75		49		39	
age (y)	67.6	1.6	72.1	1.4	77.1	1.1	82.4	2.1	67.0	1.8	72.3	1.3	77.3	1.4	83.2	1.8
MMSE	29.2	1.3	29.3	0.7	29.2	1.1	28.8	1.3	29.1	0.6	29.0	1.2	29.0	1.2	29.1	1.1
Education (y)	16.0	2.9	15.5	2.6	15.7	2.6	14.8	2.4	17.9	1.9	16.9	2.2	16.6	3.2	17.4	2.4

Volumes are presented in %.

* indicates statistical significance of comparision with the immediately preceding age group ($p < 0.05$) using pairwise t tests whose p values were adjusted by the Benjamini-Hochberg method.

MMSE, mini mental state examination; y, year.

Supplementary Table 3. Volumes of cortical and subcortical structures in the Korean elderly

	age group (female)								age group (male)							
	**65-69**		**70-75**		**75-79**		**80-84**		**65-69**		**70-75**		**75-79**		**80-84**	
	M	SD	M	SD	M	SD	M	SD	M	SD	M	SD	M	SD	M	SD
Brain volume	1031.72	66.82	995.30	69.00*	972.55	65.02*	937.19	74.03*	1129.81	81.23	1079.41	77.86*	1054.87	75.61*	1027.71	68.91*
Cortical volumes																
frontal	148.45	10.45	145.65	10.60*	144.40	10.78	139.17	9.89*	163.33	12.89	157.48	11.77*	155.27	12.26	154.01	10.05
temporal	104.46	7.64	101.06	7.83*	97.90	7.33*	95.31	7.19	113.34	8.12	108.83	8.33*	104.88	8.11*	102.65	6.34
parietal	102.39	8.23	100.03	7.69*	98.61	7.41	95.83	6.97	110.99	9.25	107.02	8.70*	105.27	9.75	103.69	7.81
occipital	46.54	4.07	44.94	4.32*	44.38	3.64	42.72	3.77*	51.28	4.77	49.01	5.02*	47.42	4.50*	45.57	4.20*
cingulate	20.79	1.97	20.46	1.96	20.11	2.03	19.16	1.60*	23.05	2.21	22.20	2.24*	22.21	2.43	21.27	2.08*
insular	11.60	0.97	11.43	0.97	11.46	0.85	11.09	0.95	12.64	0.96	12.49	1.07	12.46	1.18	12.39	1.04
Subcortical volumes																
ventricle	23.77	8.93	29.46	10.78*	34.86	12.89*	37.92	12.61	33.95	11.92	37.91	13.21*	41.33	13.95	51.70	15.10*
thalamus	14.18	1.31	13.61	1.29*	13.58	1.44	13.45	1.52	15.14	1.46	14.33	1.38*	14.22	1.45	14.05	1.19
putamen	9.41	1.07	8.92	1.11*	8.64	1.12*	8.17	1.28*	9.89	1.33	9.52	1.14*	9.30	1.23	8.75	1.33*
hippocampus	8.36	0.69	7.97	0.76*	7.56	0.75*	6.98	0.75*	8.71	0.84	8.17	0.83*	7.71	0.86*	7.45	0.62
caudate	6.61	0.73	6.45	0.73*	6.40	0.67	6.25	0.66	6.94	0.81	6.83	0.80	6.74	0.78	6.69	0.93
amygdala	3.19	0.30	3.06	0.35*	2.96	0.35*	2.78	0.34*	3.48	0.37	3.31	0.36*	3.11	0.37*	3.00	0.33
pallidus	2.71	0.38	2.68	0.44	2.75	0.38	2.77	0.36	2.92	0.45	2.89	0.37	2.93	0.49	2.95	0.46
accumbens	0.88	0.16	0.80	0.14*	0.75	0.14*	0.67	0.12*	0.91	0.14	0.87	0.15*	0.83	0.17	0.75	0.16*
n	233		239		132		34		83		128		106		53	
age (y)	67.7	1.4	72.5	1.4	76.8	1.3	81.9	1.5	67.9	1.3	72.5	1.4	77.3	1.4	82.5	1.7
MMSE	27.6	1.8	27.1	2.1	26.6	2.4	25.1	2.9	28.4	1.2	28.0	1.6	27.6	1.6	27.0	1.6
Education (y)	9.4	4.0	8.5	3.8	7.2	4.0	5.8	4.1	13.0	4.1	12.3	4.0	11.9	4.0	11.6	3.7

Volumes are presented in cm^3.

* indicates statistical significance of comparision with the immediately preceding age group ($p < 0.05$) using pairwise t tests whose p values were adjusted by the Benjamini-Hochberg method.

MMSE, mini mental state examination; y, year.

Supplementary Table 4. Volumes of cortical and subcortical structures in the Caucasian elderly

	age group (female)								age group (male)							
	**65-69**		**70-75**		**75-79**		**80-84**		**65-69**		**70-75**		**75-79**		**80-84**	
	M	SD	M	SD	M	SD	M	SD	M	SD	M	SD	M	SD	M	SD
Brain volume	999.64	91.59	955.66	89.79	931.34	80.42	900.71	75.04	1084.68	80.58	1047.76	89.74	1043.13	72.16	1000.86	91.77*
Cortical volumes																
frontal	141.71	12.30	136.48	11.54	135.06	13.11	130.14	13.30	153.72	15.53	147.02	15.22	147.70	12.02	143.79	14.39
temporal	97.88	8.82	90.57	9.04*	90.05	9.30	84.47	8.31*	106.70	9.85	99.90	10.04*	99.69	7.28	96.14	10.32
parietal	93.20	8.15	87.13	9.39*	86.08	9.47	82.94	8.85	99.29	10.26	93.98	9.51	92.63	8.50	92.20	10.58
occipital	44.28	4.91	40.44	4.55*	39.19	3.94	37.45	5.08	47.73	6.43	43.45	5.00*	43.55	4.35	42.61	4.86
cingulate	19.87	2.49	18.95	1.93	18.89	1.93	18.01	1.84	21.10	2.17	20.76	2.25	20.62	2.09	21.09	2.48
insular	11.22	1.43	10.82	1.04	10.86	1.17	10.39	0.90	12.49	1.18	11.55	1.14*	11.97	1.21	12.05	1.35
Subcortical volumes																
ventricle	28.57	16.89	35.12	19.13	39.01	15.09	45.62	21.21	33.97	12.58	41.14	17.72	57.80	28.44*	57.87	21.52
thalamus	13.20	1.55	12.07	1.28*	11.98	1.17	11.50	0.91	13.46	1.77	13.11	1.49	13.02	1.29	12.72	1.62
putamen	9.76	1.66	9.04	0.98*	8.75	1.02	9.20	1.31	10.35	1.27	9.43	1.07*	9.61	1.18	9.43	1.12
hippocampus	7.80	0.87	7.04	1.05*	6.77	0.86	6.01	0.86*	8.17	0.92	7.63	0.98*	6.98	0.89*	6.95	1.02
caudate	6.98	1.13	6.73	1.01	6.80	0.92	7.02	1.15	7.13	1.00	7.11	0.98	7.36	1.09	7.19	1.11
amygdala	3.13	0.45	2.61	0.52*	2.59	0.51	2.38	0.48	3.38	0.45	2.95	0.46*	2.84	0.44	2.84	0.54
pallidus	2.64	0.40	2.71	0.33	2.61	0.32	2.65	0.34	2.78	0.33	2.86	0.32	2.92	0.30	2.80	0.34
accumbens	1.02	0.18	0.84	0.23*	0.83	0.18	0.78	0.18	1.12	0.19	0.88	0.19*	0.86	0.18	0.82	0.20
n	23		65		51		24		16		75		49		39	
age (y)	67.6	1.6	72.1	1.4	77.1	1.1	82.4	2.1	67.0	1.8	72.3	1.3	77.3	1.4	83.2	1.8
MMSE	29.2	1.3	29.3	0.7	29.2	1.1	28.8	1.3	29.1	0.6	29.0	1.2	29.0	1.2	29.1	1.1
Education (y)	16.0	2.9	15.5	2.6	15.7	2.6	14.8	2.4	17.9	1.9	16.9	2.2	16.6	3.2	17.4	2.4

Volumes are presented in cm^3.

* indicates statistical significance of comparision with the immediately preceding age group ($p < 0.05$) using pairwise t tests whose p values were adjusted by the Benjamini-Hochberg method.

MMSE, mini mental state examination; y, year.

Supplementary Table 5. Differences of aging slopes between *APOE* ε4 carriers and non-carriers

	**Female**						**Male**					
	**Caucasian**			**Korean**			**Caucasian**			**Korean**		
	Δslope	95% CI		Δslope	95% CI		Δslope	95% CI		Δslope	95% CI	
Brain volume	**-0.610**	[-1.732,	-0.054]	-0.186	[-0.416,	0.032]	-0.122	[-0.413,	0.176]	-0.206	[-0.596,	0.216]
Cortical volumes												
frontal	-0.589	[-1.588,	0.263]	-0.158	[-0.481,	0.126]	-0.508	[-0.990,	0.005]	-0.189	[-0.631,	0.205]
temporal	-0.744	[-1.520,	0.143]	0.069	[-0.219,	0.373]	**-0.629**	[-1.104,	-0.145]	-0.161	[-0.685,	0.246]
parietal	-0.749	[-1.479,	0.045]	-0.069	[-0.414,	0.282]	-0.388	[-0.934,	0.202]	-0.269	[-0.786,	0.245]
occipital	-0.966	[-1.862,	0.060]	-0.201	[-0.602,	0.215]	-0.242	[-0.885,	0.390]	-0.367	[-1.024,	0.323]
cingulate	-0.431	[-1.527,	0.505]	-0.114	[-0.534,	0.274]	-0.395	[-0.937,	0.154]	-0.192	[-1.113,	0.571]
insular	**-0.909**	[-1.926,	-0.018]	0.270	[-0.096,	0.623]	**-0.779**	[-1.510,	-0.050]	-0.292	[-0.741,	0.211]
Subcortical volumes												
ventricle	1.411	[-2.023,	4.695]	0.151	[-2.139,	2.461]	0.006	[-1.987,	2.067]	-0.733	[-3.295,	1.634]
thalamus	-0.600	[-2.001,	0.257]	-0.246	[-0.801,	0.335]	0.145	[-0.420,	0.749]	-1.085	[-2.099,	0.037]
putamen	**-1.415**	[-2.449,	-0.313]	0.128	[-0.545,	0.850]	-0.622	[-1.359,	0.082]	0.980	[-0.066,	2.149]
hippocampus	-0.728	[-2.055,	0.465]	0.331	[-0.154,	0.786]	-0.499	[-1.305,	0.320]	-0.437	[-1.394,	0.567]
caudate	**-1.177**	[-2.471,	-0.125]	-0.235	[-0.755,	0.277]	-0.581	[-1.362,	0.341]	-0.294	[-1.317,	0.749]
amygdala	-1.037	[-2.979,	0.611]	-0.136	[-0.608,	0.432]	-0.780	[-1.940,	0.305]	0.031	[-0.949,	0.967]
pallidus	**-1.309**	[-2.179,	-0.460]	-0.038	[-0.829,	0.709]	**-0.839**	[-1.487,	-0.192]	0.072	[-0.890,	1.006]
accumbens	**-1.825**	[-3.555,	-0.154]	0.154	[-0.760,	1.050]	-0.643	[-2.100,	0.922]	-0.045	[-1.472,	1.977]

Δslope is the slope of *APOE* ε4 carriers minus the slope of non-carriers. Values are presented in % per year, and based on 10,000 iterations of linear regression analysis.

Δslope is presented in bold if the 95% confidence interval (CI) does not include the value of zero.
